# Intrathecal morphine versus femoral nerve block for pain control after total knee arthroplasty: a meta-analysis

**DOI:** 10.1186/s13018-017-0621-0

**Published:** 2017-08-16

**Authors:** Yi Tang, Xu Tang, Qinghua Wei, Hui Zhang

**Affiliations:** Department of Orthopedics, People’s Hospital of JianYang, No. 180, Yiyuan Road, Jiancheng zhen, Jianyang, Sichuan Province China

**Keywords:** Intrathecal morphine, Total knee arthroplasty, Femoral nerve block, Meta-analysis

## Abstract

**Background:**

This meta-analysis aims to illustrate the efficacy and safety of intrathecal morphine (ITM) versus femoral nerve block (FNB) for pain control after total knee arthroplasty (TKA).

**Methods:**

In April 2017, a systematic computer-based search was conducted in PubMed, EMBASE, Web of Science, Cochrane Database of Systematic Reviews, Cami Info. Inc., Casalini databases, EBSCO databases, Verlag database and Google database. Data on patients prepared for TKA surgery in studies that compared ITM versus FNB for pain control after TKA were collected. The main outcomes were the visual analogue scale (VAS) at 6, 12, 24, 48 and 72 and total morphine consumption at 12, 24 and 48 h. The secondary outcomes were complications that included postoperative nausea and vomiting (PONV) and itching. Stata 12.0 was used for pooling the data.

**Results:**

Five clinical studies with a total of 225 patients (ITM group = 114, FNB group = 111) were ultimately included in the meta-analysis. The results revealed that the ITM group was associated with a reduction of VAS at 6, 12, 24, 48 and 72 h and total morphine consumption at 12, 24 and 48 h. There was no significant difference between the occurrences of PONV. However, the ITM group was associated with an increased occurrence of itching after TKA.

**Conclusions:**

Some immediate analgesic efficacy and opioid-sparing effects were obtained with the administration of ITM when compared with FNB. The complications of itching in the ITM group were greater than in the FNB group. The sample size and the quality of the included studies were limited. A multi-centre RCT is needed to identify the optimal method for reaching maximum pain control after TKA.

**Electronic supplementary material:**

The online version of this article (doi:10.1186/s13018-017-0621-0) contains supplementary material, which is available to authorized users.

## Background

Total knee arthroplasty (TKA) leads to considerable postoperative pain [1, 2]. Ineffective pain control after TKA can cause many side effects [3]. Optimal pain control can not only decrease complications but also facilitate fast recovery during the immediate postoperative period [4]. Currently, a multimodal technique, a new concept, is accepted by most surgeons. Multimodal analgesia includes regional techniques, systemic opioids and preoperative administration gabapentin. Thus, femoral nerve block (FNB) and intrathecal morphine (ITM) are seldom used alone for the management of postoperative pain, though they are known to provide excellent analgesia [5]. Since the recommendation by the PROSPECT working group [6], FNB and ITM have become two common methods for postoperative analgesia management following TKA.

Regarding the efficacy and safety of ITM and FNB for pain control after TKA, there has yet to be a consensus. Recently, Li et al. [7] conducted a meta-analysis for this topic. However, several disadvantages existed in that meta-analysis. First it ignored important randomized controlled trials (RCTs), which may have had an important influence on the final results [8]. Furthermore, continuous FNB and single-shot FNB were not compared in a subgroup analysis. Thus, the purpose of this meta-analysis is to compare the efficacy and safety of ITM and FNB for pain control after TKA (Table 1).

**Table 1:** The general characteristic of the included studies.

Study	Country	FNB Group			Surgery	ITM Group			Outcomes	Follow-up
		No. of patients	Method	Drug		No. of patients	Dose	Concomitant pain management		
Frassanito 2010	Italy	26	SFNB	Ropivacaine 0.75% 25 ml	TKA	26	Hyperbaric bupivacaine 15 mg plus 0.1 mg of morphine sulphate	Paracetamol 1 g i.v. four times daily and intravenous. Ketorolac 30 mg 2 times every 24 h	1, 2, 3, 4	48 h
Mohamed 2016	Egypt	20	SFNB	Hyperbaric bupivacaine 15 mg	TKA	20	0.2 mg morphine	PCA with morphine	1, 2, 3, 4	3 month
Olive 2015	Australia	27	CFNB	20 ml bottle of 0.5% bupivacaine	TKA	28	0.5% bupivacaine 3.5 ml plus 0.175 mg morphine	0.5% bupivacaine 3.5 mL	1, 2, 3, 4	1 year
Sites 2004	USA	20	SFNB	40 mL of 0.5% ropivacaine with 75 mg of clonidine and 5 mg/mL of epinephrine	TKA	20	0.25 mg morphine and 15 mg hyperbaric bupivacaine	30 mg ketorolac IV every 6 h	1, 2, 3, 4	48 h
Tarkkila 1998	India	18	CFNB	0.25% bupivacaine at a rate of 0.1 mL kg 1 h 1	TKA	20	0.3 mg morphine mixed with bupivacaine	Oxycodone 0.1–0.14 mg/kg	1, 2, 3, 4	48 h

*FNB* femoral nerve block, *ITM* intrathecal morphine, *TKA* total knee arthroplasty

## Methods

This systematic review was reported according to the preferred reporting items for systematic reviews and meta-analyses (PRISMA) guidelines [9].

### Search strategies

The following databases were searched in September 2016 without restrictions on location or publication types: PubMed (1950–April 2017), EMBASE (1974–April 2017), the Cochrane Library (April 2017, Issue 4), Cami info. Lnc (1950–April 2017), Casalini databases (1950–April 2017), EBSCO databases (1950–April 2017), Verlag database (1950–April 2017) and Google database (1950–April 2017). The Mesh terms and their combinations used in the search were as follows: “analgesia” OR “pain management” OR “anaesthetic agents” OR “total knee arthroplasty” OR “total knee replacement” OR “TKA” OR “TKR” OR ““Arthroplasty, Replacement, Knee”[Mesh]” AND “intrathecal morphine” OR “femoral nerve block”. The reference lists of related reviews and original articles were searched for any relevant studies, including RCTs involving adult humans. There was no language restriction for this meta-analysis. When multiple reports describing the same sample were published, the most recent or complete report was used. This meta-analysis gathered data from published articles, and thus, no ethics approval was necessary for this article.

### Inclusion criteria and study selection

**Fig. 1:**
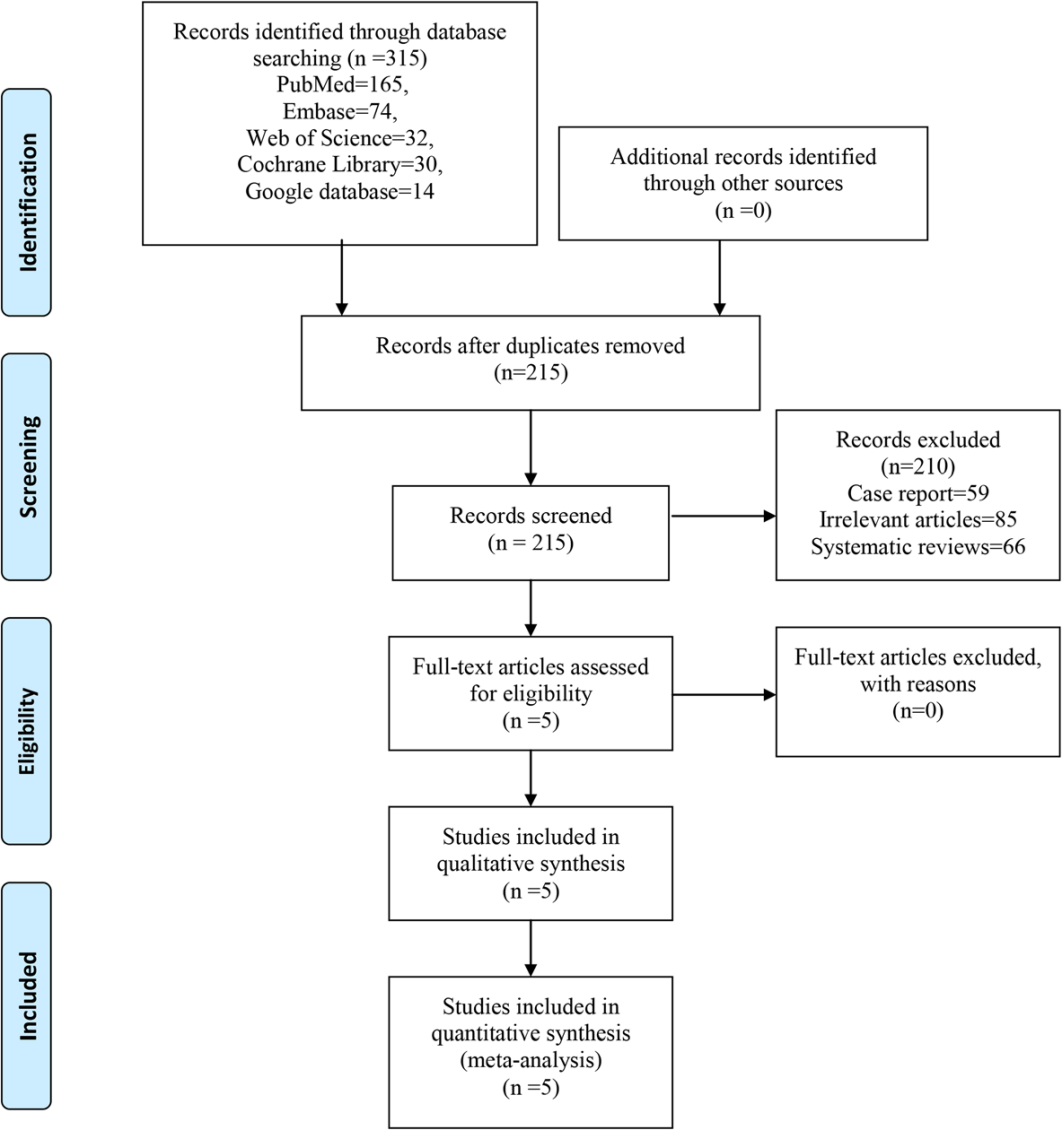
Flowchart of study search and inclusion criteria

Patients: patients prepared for primary unilateral TKA; Intervention: use ITM as an intervention group; Comparison: use FNB as a comparison group; Outcomes: VAS at 6, 12, 24, 48 and 72 h, total morphine consumption at 12, 24 and 48, and related complications (postoperative nausea and vomiting (PONV) and itching); Study design: RCTs (Fig. 1).

Two independent reviewers screened the title and abstracts of the identified studies after removing the duplicates from the search results. Any disagreements about the inclusion or exclusion of a study were solved through discussion or consultation with an expert (skilled in pain control and TKA). The reliability of the study selection was determined by Cohen’s kappa test, and the acceptable threshold value was set at 0.61 [6, 7].

### Data abstraction and quality assessment

A specific extraction was conducted to collect data in a pre-generated standard Microsoft^®^ Excel (Microsoft Corporation, Redmond, Washington, USA) file. The items extracted from relevant studies were as follows: first author and publication year, country, sample size of the intervention and control groups, preoperative and postoperative doses, timing and frequency and the total dose of gabapentin per number of days and follow-ups. Abstracted and recorded in the spreadsheet were outcomes such as the VAS at 6, 12, 24, 48 and 72 h; total morphine consumption at 12, 24 and 48 h; and related complications (PONV and itching). Postoperative pain intensity was measured using a 110-point VAS (0 = no pain and 100 = extreme pain). When the numerical rating scale (NRS) was reported, it was converted to a VAS. Additionally, a 10-point VAS was converted to a 100-point VAS [10]. Data in other forms (i.e. median, interquartile range, and mean ± 95% confidence interval (CI)) were converted to the mean ± standard deviation (SD) according to the Cochrane Handbook [11]. If the data were not reported numerically, we extracted these data using the “GetData Graph Digitizer” software from the published figures. All the data were extracted by two independent reviewers, and disagreements were resolved through discussion.

The quality of all included trials was independently assessed by two reviewers on the basis of the Cochrane Handbook for Systematic Reviews of Interventions, version 5.1.0 (http://training.cochrane.org/handbook/) [11]. A total of seven domains were used to assess the overall quality: random sequence generation, allocation concealment, blinding of participant and personnel, blinding of outcome assessment, incomplete outcome data, selective reporting and other bias. Each domain was measured as low bias, unclear bias or high bias.

### Outcome measures and statistical analysis

Continuous outcomes (VAS at 6, 12, 24, 48 and 72 h and total morphine consumption at 12, 24 and 48 h) were expressed as weighted mean differences (WMD) with 95% CI. Dichotomous outcomes (the occurrence of PONV and itching) were expressed as a risk ratio (RR) with 95% CI. Statistical significance was set at *P* < 0.05 to summarize the findings across the trials. Variables in the meta-analysis were calculated using Stata software, version 12.0 (Stata Corp., College Station, TX). Statistical heterogeneity was evaluated using the chi-square test and the *I* ^2^ statistic. When there was no statistical evidence of heterogeneity (*I* ^2^ < 50%, *P* > 0.1), a fixed-effects model was adopted; otherwise, a random-effects model was chosen. Publication bias was tested using funnel plots. Publication bias was visually assessed using funnel plots and was quantitatively assessed using Begg’s test. Subgroup analysis was conducted according to the type of continuous FNB (CFNB) or single shot FNB (SFNB). We did not perform the publication bias since the numbers were less than ten.

## Results

### Search results and quality assessment

In the initial search, a total of 386 studies were identified from the electronic databases (PubMed = 165, EMBASE = 74, Web of Science = 32, Cochrane Database of Systematic Reviews = 30, Google database = 14). Then, all papers were input into Endnote X7 (Thomson Reuters Corp., USA) software for the removal of duplicate papers. A total of 308 papers were reviewed, and 209 papers were removed according to the inclusion criteria at abstract and title levels. Ultimately, five clinical studies with 225 patients (ITM group = 114, FNB group = 111) were included in this meta-analysis [8, 12–15].

**Fig. 2:**
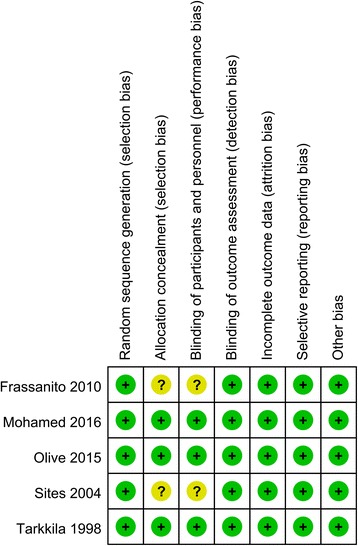
The risk of bias graph

**Fig. 3:**
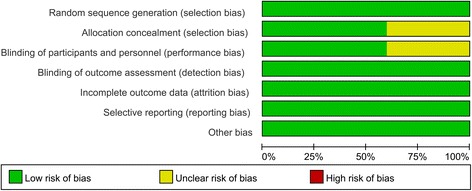
Risk of bias summary of included randomized controlled trials. +, no bias; −, bias;?, bias unknown

The quality assessment of the included studies is summarized in Figs. 2 and 3. All studies describe the random sequence generation procedure. Two studies did not describe allocation concealment and the blinding of participants and personnel and thus had an unclear risk of bias. The rest of the items all had a low risk of bias. The overall kappa value for the evaluation of the risk of bias of included RCTs was 0.763, indicating that the agreement between the two reviewers was acceptable.

### VAS at 6, 12, 24, 48 and 72 h

**Fig. 4:**
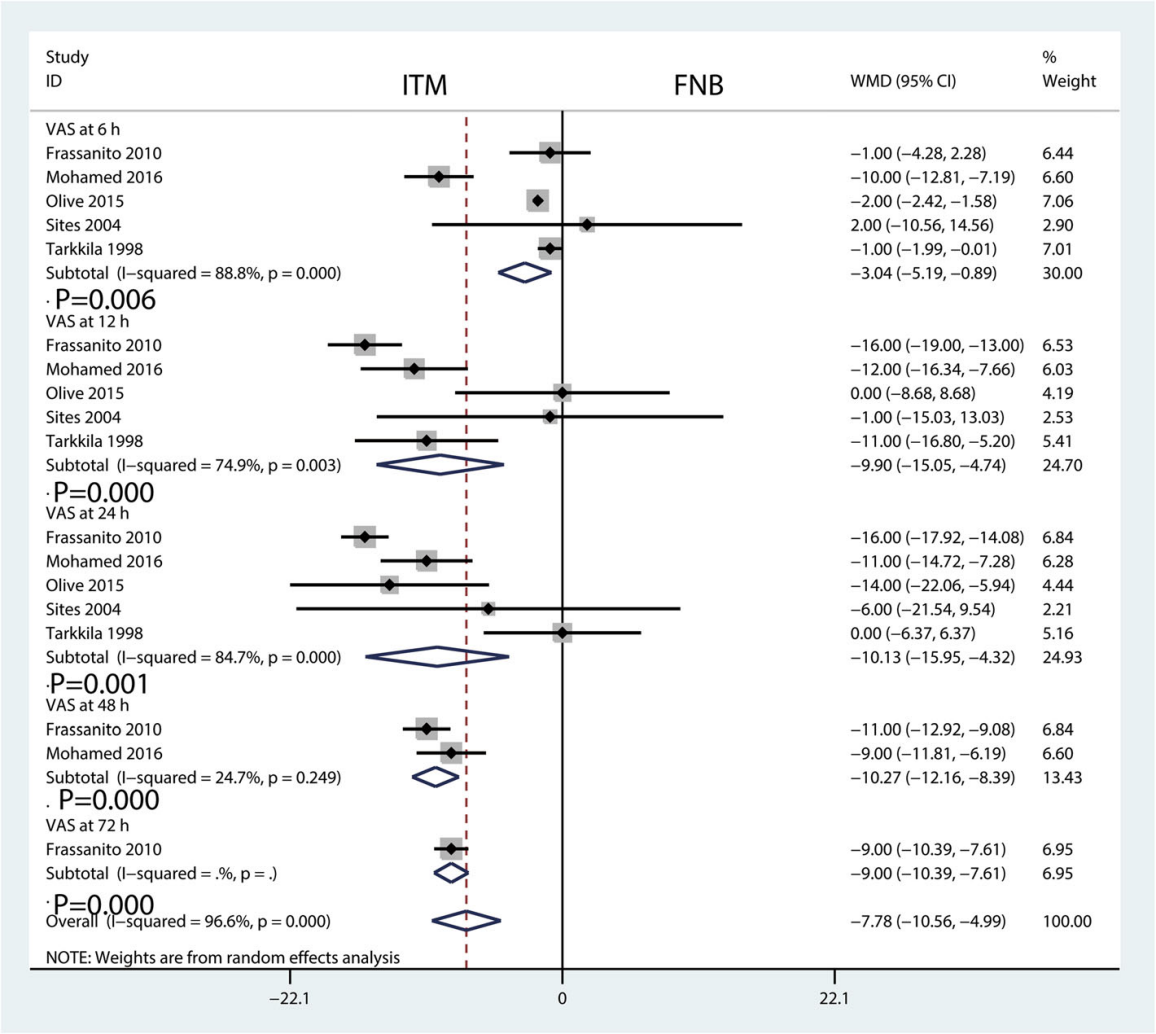
Forest plots of the included studies comparing the VAS at 6, 12, 24, 48 and 72 h

Postoperative VAS scores at 6 h were reported in three studies, and the pooled results indicated that ITM can decrease VAS scores at 6 h (WMD = −3.04, 95% CI −5.19, −0.89, *P* = 0.006, Fig. 4) when compared with the FNB group. Postoperative VAS scores at 6 h in the included studies had a large heterogeneity (*I* ^2^ = 88.8%, *P* = 0.000), which required a random-effects model that was performed to analyse the data.

The meta-analysis results indicated that ITM can decrease VAS scores at 12 h (WMD = −9.90, 95% CI −15.05, −4.74, *P* = 0.000, Fig. 4) when compared with the FNB group. Postoperative VAS scores at 12 h in the included studies had a large heterogeneity (*I* ^2^ = 74.9%, *P* = 0.003), which required a random-effects model that was performed to analyse the relevant data.

The results of the meta-analysis indicated that ITM can decrease VAS scores at 24 h (WMD = −10.27, 95% CI −12.16, −8.39, *P* = 0.001, Fig. 4) when compared with the FNB group. Postoperative VAS scores at 24 h in the included studies had a large heterogeneity (*I* ^2^ = 84.7%, *P* = 0.000), which required a random-effects model that was performed to analyse the relevant data.

The results of the meta-analysis indicated that ITM can decrease VAS scores at 48 h (WMD = −10.27, 95% CI −12.16, −8.39, *P* = 0.000, Fig. 4) compared with the FNB group. Postoperative VAS scores at 48 h in the included studies had little heterogeneity (*I* ^2^ = 24.7%, *P* = 0.249).

The results of the meta-analysis indicated that ITM can decrease VAS scores at 72 h (WMD = −9.00, 95% CI −10.39, −7.61, *P* = 0.000, Fig. 4) when compared with the FNB group.

### Total morphine consumption at 12, 24 and 48 h

**Fig. 5:**
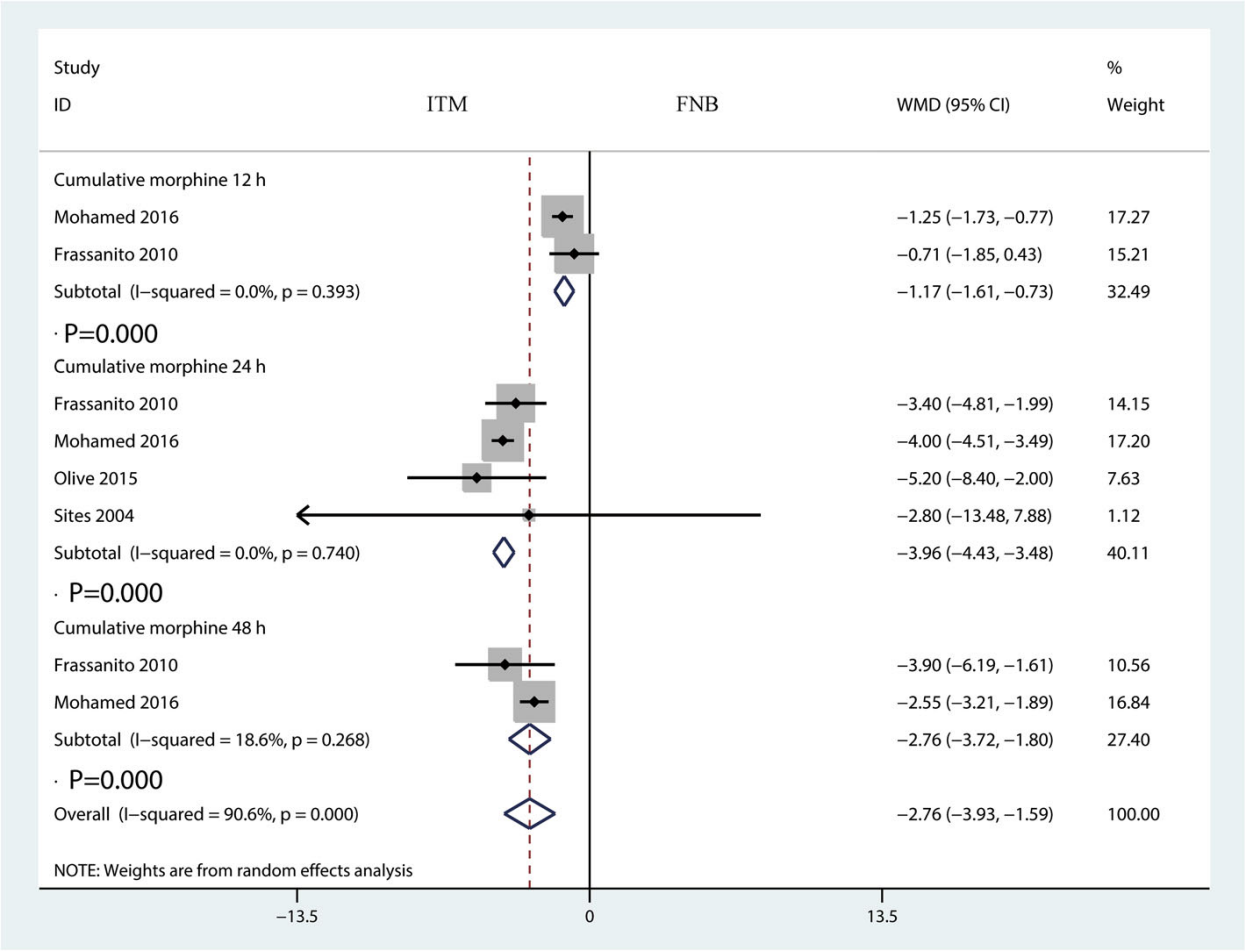
Forest plots of the included studies comparing the total morphine consumption at 12, 24 and 48h

Total morphine consumption was presented in six studies. The pooled results indicated that ITM can reduce total morphine consumption at 12 h (WMD = −1.17, 95% CI −1.61, −0.73, *P* = 0.000, Fig. 5), 24 h (WMD = −3.96, 95% CI −4.43, −3.48, *P* = 0.000, Fig. 5) and 48 h (WMD = −2.76, 95% CI −3.72, −1.80, *P* = 0.000, Fig. 5).

### The occurrence of PONV

**Fig. 6:**
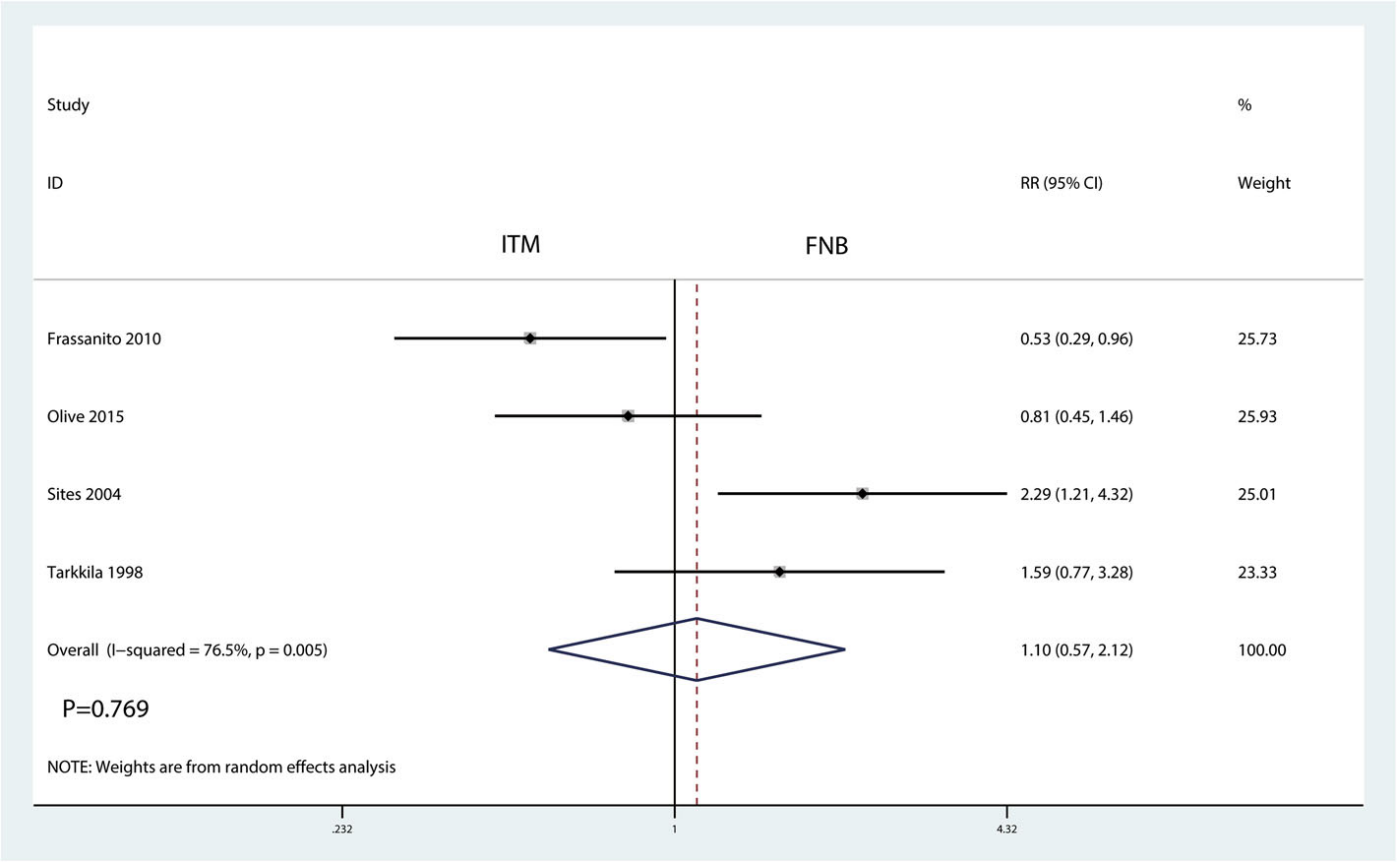
Forest plots of the included studies comparing the occurrence of PONV

There were no significant differences between the groups in the occurrence of PONV (RR = 1.10, 95% CI 0.57, 2.12, *P* = 0.769, Fig. 6). There was high heterogeneity between the included studies (*I* ^2^ = 76.5%, *P* = 0.005), and thus, a random-effect model was performed.

### The occurrence of itching

**Fig. 7:**
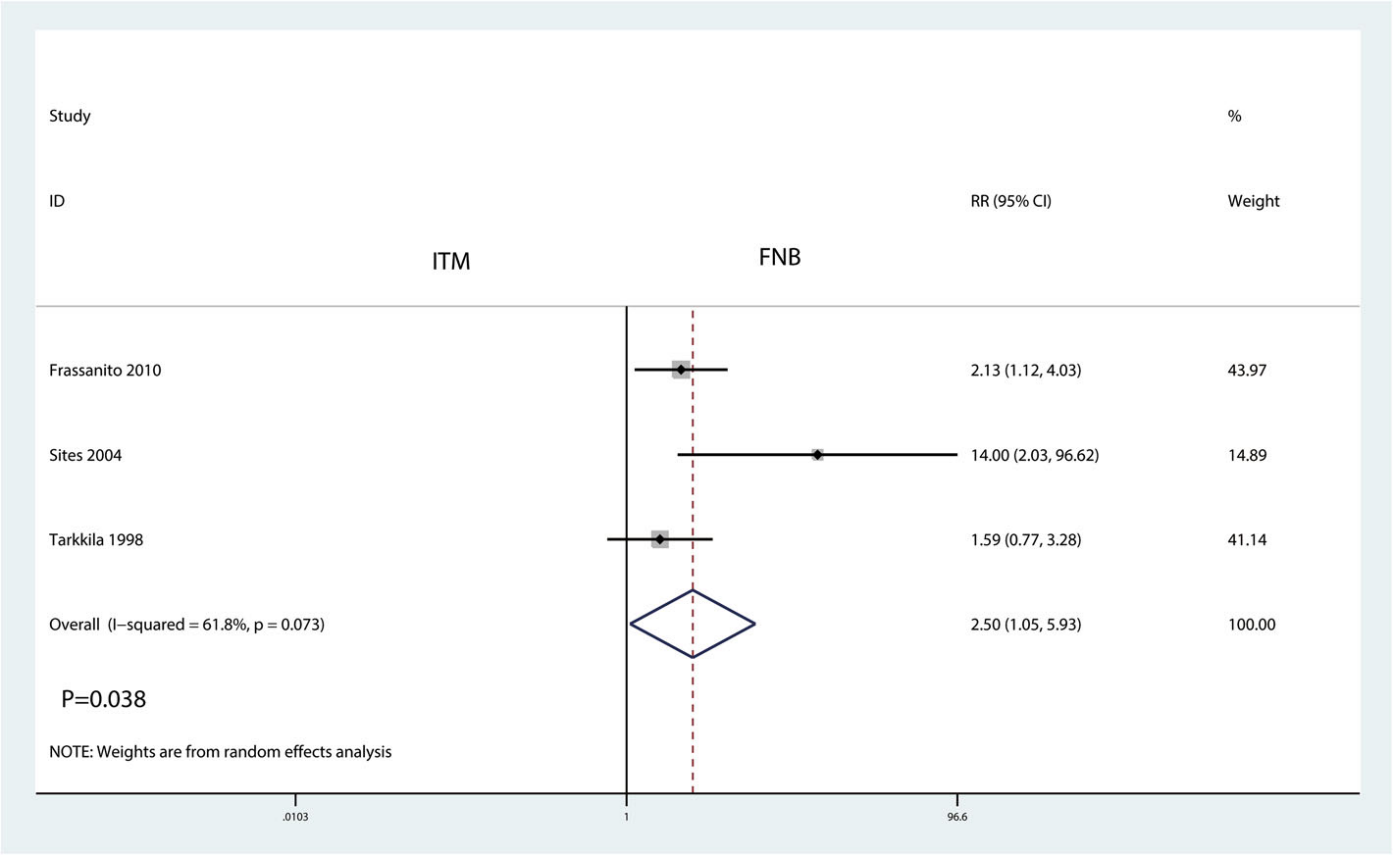
Forest plots of the included studies comparing the occurrence of itching

The occurrence of itching was presented in three studies. Compared with the FNB group, ITM was associated with an increase of the occurrence of itching (RR = 2.50, 95% CI 1.05, 5.93, *P* = 0.038, Fig. 7). There was a high heterogeneity (*I* ^2^ = 61.8%, *P* = 0.073) among the included studies, and thus, a random-effects model was performed.

### Subgroup analysis and dose-response relationship

Subgroup analysis was conducted according to the type of FNB (CFNB or SFNB). The results are shown in Additional file 1 and indicate that there was no significant difference between the CFNB and SFNB in terms of VAS at 6, 12 and 24 h.

## Discussion

This meta-analysis aimed to illustrate the optimal method of pain control in TKA patients using ITM and FNB. Pooled results indicated that the ITM group had lower pain scores at 6, 12, 24, 48 and 72 h after TKA. Furthermore, ITM was associated with less total morphine consumption at 12, 24 and 48 h after TKA. However, ITM was associated with an increase of the occurrence of itching. There was no significant difference in the occurrence of PONV after TKA. A major strength of this meta-analysis was that we comprehensively searched the electronic databases and used a rigorous statistical calculation. A total of five relevant studies were included, and the risk of bias was relatively high.

TKA is characterized by severe postoperative pain and morphine-related complications. A meta-analysis has identified that the administration of FNB was associated with a reduction in pain intensity after TKA [5]. FNB has been identified as an effective method for decreasing postoperative pain and morphine consumption after TKA. Alternatively, there is solid evidence supporting the use of ITM [16, 17]. Currently, there is no consensus regarding which methods are more effective after TKA surgery. Thus, we performed this meta-analysis to provide summary evidence for surgeons for a better choice of pain control following TKA. The current meta-analysis indicated that ITM was more effective than FNB at 6, 12, 24, 48 and 72 h after TKA. The doses of ITM ranged from 0.1 to 0.5 mg [18]. A meta-analysis showed that the rate of episodes of respiratory depression (doses <0.3 mg) was equal to that of the systemic opioids group [19]. The dose of morphine in the ITM group in the meta-analysis ranged from 0.1 to 0.3 mg, and this may be due to heterogeneity in the sources of different studies. Meanwhile, two studies performed the CFNB, and the remaining three studies performed the SFNB.

Since pain intensity was decreased in the ITM group, morphine consumption as a supplement for pain control was decreased correspondingly. Pooled results indicated that the ITM group was associated with a reduction of total morphine consumption at 12 h by 1.17 mg (WMD = −1.17, 95% CI −1.61, −0.73, *P* = 0.000) and 3.96 mg at 24 h (WMD = −3.96, 95% CI −4.43, −3.48, *P* = 0.000) and 48 h (WMD = −2.76, 95% CI −3.72, −1.80, *P* = 0.000). Li et al. [7] found that total morphine consumption in the ITM group was less than in the FNB group, at appropriately 0.84 mg. Chang et al. [20] reported that the addition of IT morphine 0.1 mg to continuous femoral 3-in-1 nerve block improves postoperative analgesia and morphine consumption after TKA.

Another issue that must be addressed is that FNB requires the use of an ultrasound machine and a special needle, which add to the cost. Additionally, another anaesthetist is needed to perform FNB, and thus, it may add to the total operation time. In this regard, ITM is very cost effective, time-saving and a relatively simple technique.

There were a total of six limitations in this meta-analysis: (1) only five RCTs were included, which may have affected the precision of the effect size estimations; (2) follow-up in the included studies ranged from 24 h to 6 months, and the relatively short-term follow-up may underestimate the complication rate; (3) the dosage of ITM differed between the studies, and although a subgroup analysis was conducted to decrease heterogeneity, that could affect the precision of the results; (4) the dosage, drugs and type of FNB differed between the studies, which could affect the precision of the results; (5) multiple analgesic approaches differed from one another, and consistent multiple analgesic approaches are needed to identify the most effective pain control method; and (6) publication bias was not performed due to the limited number of included studies, and there was potential publication bias.

## Conclusion

In conclusion, some immediate analgesic efficacy and opioid-sparing effects were obtained with the administration of ITM when compared with FNB. Complications of itching in the ITM group were greater than in the FNB group. Because the sample size and the number of included studies were limited, a multi-centre RCT is needed to identify the effects of ITM in reducing acute pain following TKA surgery.

## Additional file

Additional file 1: Subgroup analysis results of VAS score. (DOCX 6040 kb)

## Abbreviations

CFNB: Continuous femoral nerve block
CI: Confidence interval
DVT: Deep venous thrombosis
FNB: Femoral nerve block
ITM: Intrathecal morphine
NRS: Numerical rating scale
PONV: Postoperative nausea and vomiting
PRISMA: Preferred Reporting Items for Systematic Reviews and Meta-analyses
RCTs: Randomized controlled trials
RR: Relative risk
SD: Standard deviation
SFNB: Single shot femoral nerve block
TKA: Total knee arthroplasty
VAS: Visual analogue scale
WMD: Weight mean difference
