# Supplementary material for: Intrathecal morphine versus femoral nerve block for pain control after total knee arthroplasty: a meta-analysis
Source: J Orthop Surg Res. 2017 Aug 16;12:125. doi: 10.1186/s13018-017-0621-0 (PMC5559845; doi:10.1186/s13018-017-0621-0)
Supplement: Additional file 1: — Subgroup analysis results of VAS score. (DOCX 6040 kb) [file 13018_2017_621_MOESM1_ESM.docx]

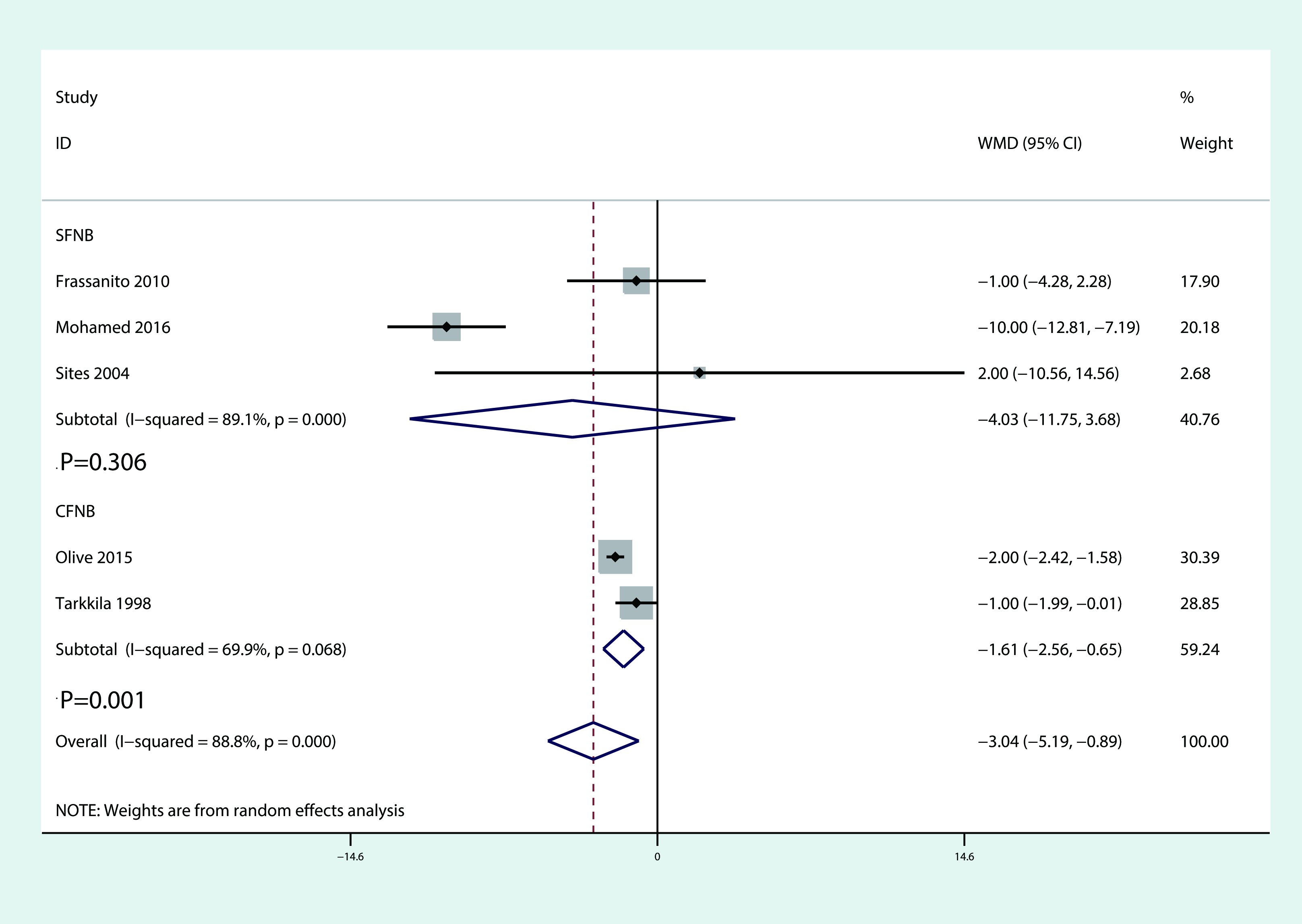


Figure S1: Subgroup analysis results of VAS score at 6 h.


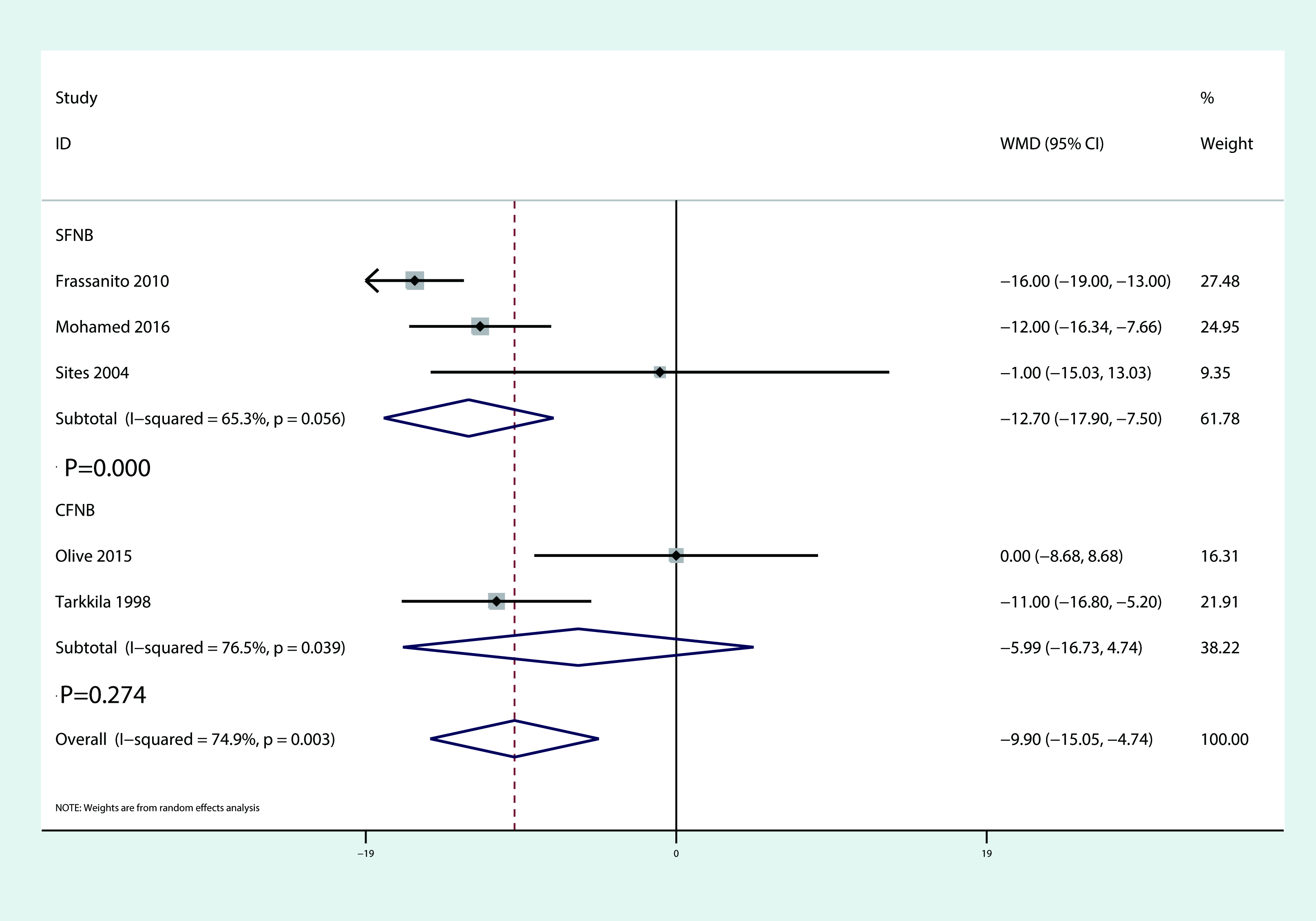


Figure S2: Subgroup analysis results of VAS score at 12 h.


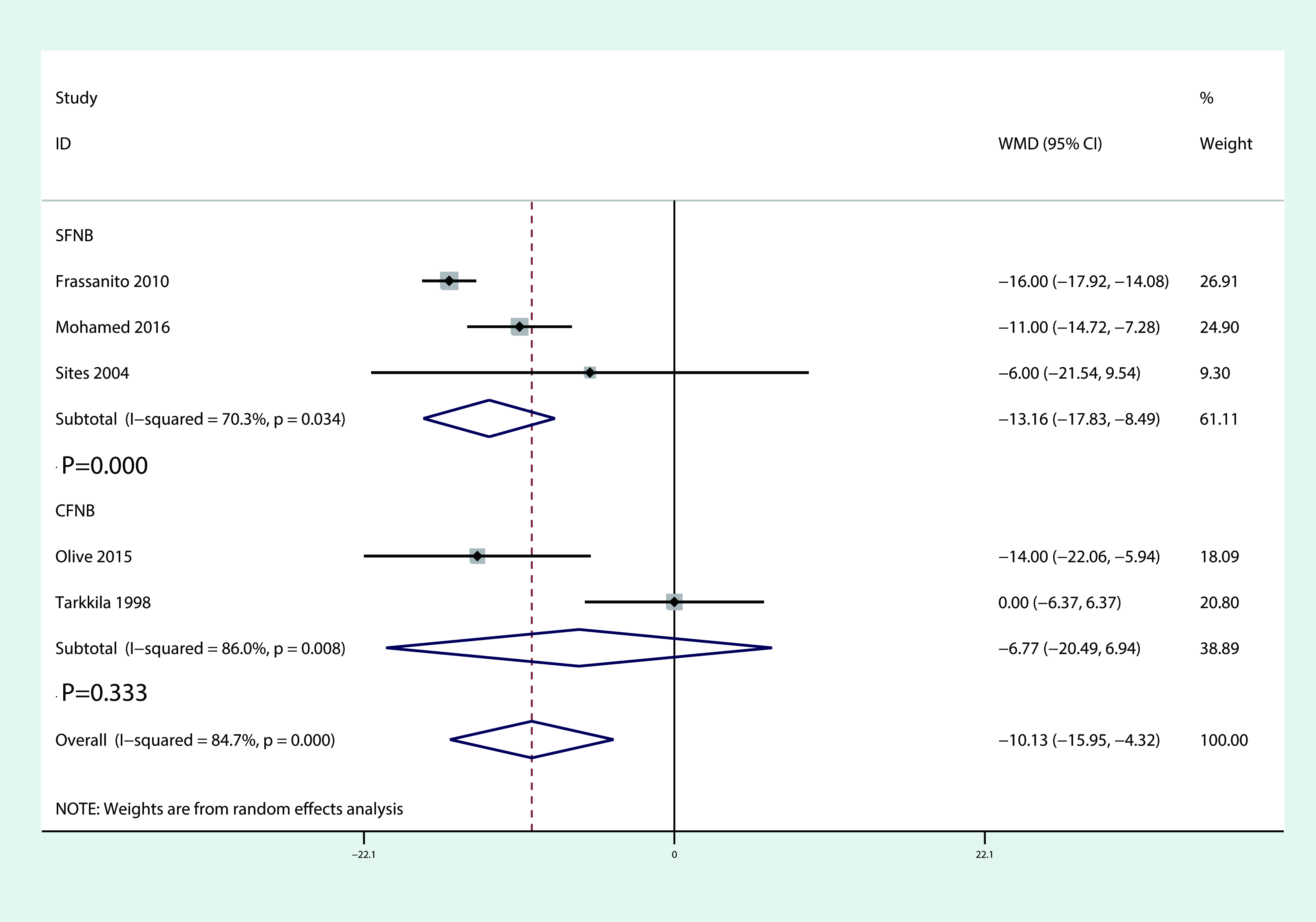


Figure S3: Subgroup analysis results of VAS score at 24 h.
